# Effect of TiO_2_ and ZnO Nanoparticles on the Performance of Dielectric Nanofluids Based on Vegetable Esters During Their Aging

**DOI:** 10.3390/nano10040692

**Published:** 2020-04-06

**Authors:** Inmaculada Fernández, Rafael Valiente, Félix Ortiz, Carlos J. Renedo, Alfredo Ortiz

**Affiliations:** 1 Department of Electrical and Energy Engineering, Faculty of Industrial and Telecommunications Engineering, University of Cantabria, 39005 Santander, Spain; felix.ortiz@unican.es (F.O.); carlos.renedo@unican.es (C.J.R.); alfredo.ortiz@unican.es (A.O.); 2 Department of Applied Physics, Science Faculty, University of Cantabria, IDIVAL, 39005 Santander, Spain; rafael.valiente@unican.es

**Keywords:** natural ester, TiO_2_, ZnO, thermal aging, nanofluid, breakdown voltage

## Abstract

Over the last few decades the insulating performance of transformer oils has been broadly studied under the point of view of nanotechnology, which tries to improve the insulating and heat dissipation performance of transformer oils by suspending nanoparticles. Many authors have analyzed the thermal and dielectric behavior of vegetable oil based-nanofluids, however, very few works have studied the evolution of these liquids during thermal aging and their stability. In this paper has been evaluated the performance of aged vegetable oil based-nanofluids, which have been subjected to accelerated thermal aging at 150 °C. Nanoparticles of TiO_2_ and ZnO have been dispersed in a commercial natural ester. Breakdown voltage, resistivity, dissipation factor and acidity of nanofluid samples have been measured according to standard methods, as well as stability. Moreover, it has been analyzed the degradation of Kraft paper through the degree of polymerization (DP). The results have showed that although nanoparticles improve breakdown voltage, they increase the ageing of insulation liquids and dielectric paper.

## 1. Introduction

Transmission and distribution transformers are one of the most critical parts of the power networks. Oil and paper are the most common materials in the insulation system of power transformers. These machines basically consist of a magnetic core and two conductive windings. These components are immersed in a tank that is filled with a liquid that performs two functions: dielectric insulation and cooling of the windings. This fluid is in most of the cases mineral oil. Apart from the dielectric fluid, in the transformer the conductors are surrounded by several layers of paper, which once impregnated with oil, behaves as an excellent dielectric material.

The transformers downsizing and the increase of their voltage have enhanced the development of new dielectric fluids [1]. In fact, many research works during last decade have reported that different nanoparticles can considerably affect dielectric and cooling properties of transformer oil [2,3].

The nanoparticles used to improve the properties of dielectric liquids are classified in three categories: conductor (Fe_3_O_4_, Fe_2_O_3_ and SiC), semiconductor (TiO_2_, ZnO, CuO and Cu_2_O) and insulators (Al_2_O_3_, SiO_2_ and BN) [2,3,4]. Different dielectric nanofluids have been analyzed by several researchers who have found excellent insulating and thermal properties, which are essential for power transformers. For instance, Ibrahim et al. [5] measured the breakdown voltage of nanofilled mineral oil using conductive nanoparticles (Fe_2_NiO_4_), semiconductor nanoparticles (CdS) and a mixture of both in two different concentrations. The results of their work showed that the breakdown voltages of analyzed nanofluids were improved. However, the increase in the concentration on nanoparticle mixture can provoke an adverse effect on the breakdown voltage. Sima et al. [6] also obtained much higher voltage levels of positive breakdown than that of pure mineral oil with other nanoparticles (Fe_3_O_4_, TiO_2_ and Al_2_O_3_). Similar results were obtained by Wang et al. for the same nanoparticles (Fe_3_O_4_, TiO_2_ and Al_2_O_3_) suspended in mineral oil [7]. Other authors have also analyzed the influence of three different nanoparticles (Fe_3_O_4_, ZnO and SiO_2_) [8]. These not only studied nanofluids based on mineral oil, but also based on two types of vegetable oils. These authors concluded that the breakdown voltage of nanofluids based on natural liquids was higher when compared with mineral oil. By considering the fire point enhancement, vegetable oils with nanoparticles obtained excellent performance compared to nanofluids based on mineral oil. Nevertheless, the viscosity of vegetable nanofluids were high compared with mineral oil with nanoparticles, this behavior could represent a disadvantage under the point of view of cooling characteristics. Nanofluids based on vegetable oils have been also studied by Li et al. [9] who dispersed Fe_3_O_4_ nanoparticles in a vegetable dielectric oil obtained in the laboratory and in two commercial vegetable oils. These authors concluded that breakdown voltages of nanofluids were higher than pure oil. Moreover, the volume resistivity of the nanofluids and the pure oil were quite similar at frequencies exceeding 100 Hz. However, the relative permittivity of the nanofluids was greater than that of the pure vegetable oil. Raymon et al. [5] who also worked with natural oils (coconut, sunflower, cottonseed, soya bean, rapeseed and rice bran) founded that the conductor (Fe_2_O_3_), semiconductor (CdS, TiO_2_) and insulator nanoparticles (Al_2_O_3_) raise the breakdown voltage of natural ester oils. Although, there are works that have analyzed the performance of vegetable oil based-nanofluids, currently most of works have prepared nanofluids using commercial mineral oils [2,3,4]. On the other hand, it should be highlighted that although dielectric properties are critical characteristics of insulating fluids, it is significantly important that nanofluids besides possess suitable cooling properties. For this reason, some authors have focused their works on these characteristics [10,11,12,13]. Their results conclude that magnetic fluids reduce temperatures at transformer winding and top oil, which would diminish the aging rate as it happens with pure dielectric liquids [14,15].

In addition to the effect of nanoparticles on transformer oil, some authors have also evaluated the properties of nanofluids during aging, in order to observe their stability during transformers’ operation. Zmarzly and Dobry [16] verified the applicability of C60 fullerenes as inhibitors of the aging process in dielectric oil. These authors aged mineral oil samples with and without fullerene at temperature of 110 °C for 144 h. They obtained that all the doped samples have lower water content than the samples without added fullerenes. Moreover, they observed that the breakdown voltage decreased more slowly for oil/fullerenes solution with a lower concentration, whereas tan δ decreases for oils with higher content of fullerenes. Mu-tian et al. [17] also carried out thermal aging, however, they aged their nanofluids (dispersed TiO_2_ nanoparticles into a mineral oil) at 130 °C for 144 h. The results of their work showed that the breakdown voltage and partial discharge inception voltage (PDIV) of aged nanofluids can be increased up to 1.08 and 1.12 times compared with pure aged oil, respectively. Emara et al. [18] also carried out accelerated aging tests with mineral oil and TiO_2_ nanoparticles. In their work, these authors exposed fresh mineral oil to accelerated aging tests at 120 °C for two different aging periods, 72 and 240 h. Then, aged oil-based nanofluids were prepared using TiO_2_ nanoparticles. The main conclusions of this work were that a remarkable increase in breakdown voltage had been obtained, as well as an important decrease of the dielectric dissipation factor. Prasath et al. [19] also added nanoparticles (ZnO and Al_2_O_3_) to pure and aged mineral oil. It was concluded that breakdown voltage, flash and fire point of pure and aged transformer oil are increased due to the nanoparticles. Furthermore, the viscosity of nanofluids did not show any drastic change as compared with pure and aged transformer oil.

Although there are different works that have studied the dielectric performance of vegetable oil based-nanofluids, currently few works have studied the evolution during thermal aging of these nanofluids. The aim of this article is to evaluate the performance of aged vegetable oil based-nanofluids, which have been subjected to accelerated thermal aging at 150 °C for 312 h. The used semiconductor nanoparticles have been TiO_2_ and ZnO. Breakdown voltage, resistivity, dissipation factor and acidity of nanofluid samples have been measured according to standard methods. Moreover, the degradation of Kraft paper through the degree of polymerization (DP) has been analyzed. The comparison of vegetable oil and nanofluids behavior was performed before and during the aging period. Unlike previous published works, this paper has evaluated the stability of nanofluids during thermal aging, which has provided useful information about nanofluids behavior at a temperature different from the ambient one, being this more suitable to evaluate the possibilities of nanofluids under conditions more similar to real working conditions. Additionally, this paper has studied the influence of very high initial moisture in the insulation liquids, on the dielectric and aging behavior, which has not been considered previously on other studies focused on nanofluids used in power transformers.

## 2. Materials and Methods

### 2.1. Materials

The base oil used was a commercially available vegetable oil with the specifications described in Table 1. The TiO_2_ and ZnO nanoparticles used were also commercially available, their characteristics are gathered in Table 2.

**Figure 1:**
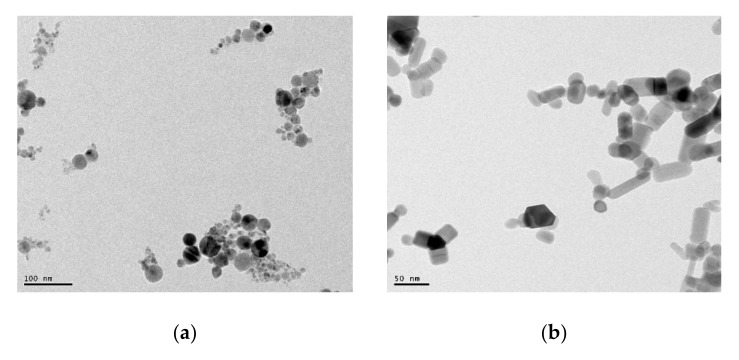
TEM micrographs of (**a**) TiO_2_ nanoparticles and (**b**) ZnO nanoparticles.

The Figure 1 demonstrates the microscopy images of the nanoparticles captured by TEM. These micrographs revealed that the average sizes of the two nanoparticles were 60 nm (ZnO) and 45 nm (TiO_2_).

**Figure 2:**
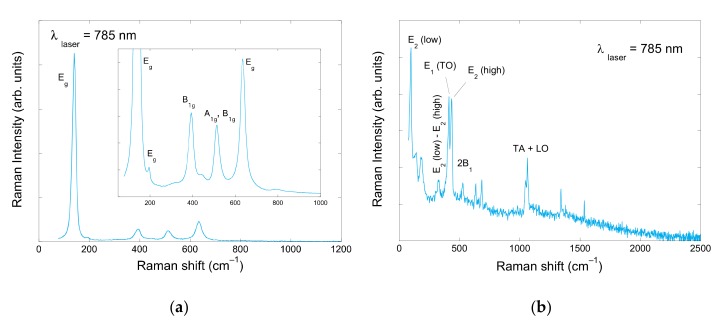
Raman spectra of (**a**) TiO_2_ nanoparticles and (**b**) ZnO nanoparticles.

Additionally, the unpolarized Raman spectra corresponding to TiO_2_ and ZnO nanocrystals were obtained using a Raman Glacier X (BWTEK) spectrometer (Newark, DE, USA) equipped with a thermoelectrically cooled CCD array under 785 nm excitation, as it can be observed in the Figure 2.

**Figure 3:**
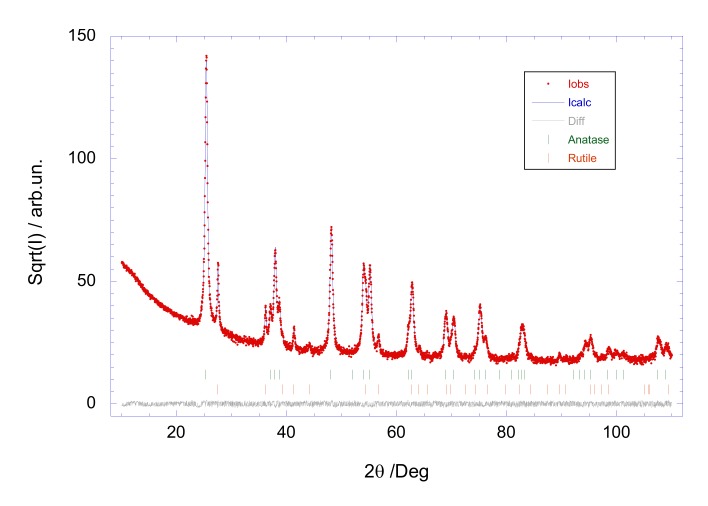
Rietveld refinement of the TiO_2_ X-ray diffraction using radiation from a Cu tube (λ = 1.5418 Å).

**Figure 4:**
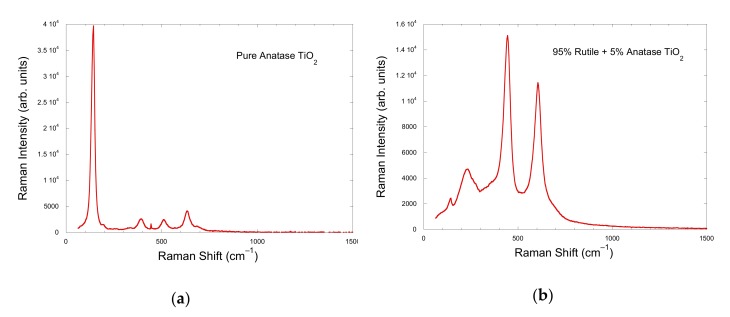
Raman spectra of (**a**) Anatase TiO_2_ phase and (**b**) 95% Rutile and 5% Anatase TiO_2_ phase.

Raman spectroscopy is a non-destructive research tool, generally used as a probe providing chemical and structural information. The Raman spectrum of Figure 2a corresponds mainly to the anatase TiO_2_ phase, where the peaks at 141, 195, 394, 513 and 635 cm^–1^ were assigned to E_g_, E_g_, B_1g_, A_1g_ + B_1g_ and E_g_ according to Swamy et al. [20]. The small peak at 445 cm^–1^ is characteristic to rutile TiO_2_ [21]. This spectrum is characteristic to TiO_2_ Degussa P-25 with a ratio anatase-rutile of about 3 to 1. However, the Rietveld refinement of X-Ray pattern diffraction (Figure 3) together with the intensity analysis of the individual Raman spectrum of anatase and rutile TiO_2_ phases (Figure 4), point out to 90% anatase and 10% rutile composition for the TiO_2_ nanoparticles used in this study. Whereas the main features of the Raman spectrum of ZnO (Figure 2b) could be ascribed to wurtzite ZnO, where the most intense Raman peaks corresponded to the E_2_ (low) mode at 99 cm^–1^ and E_2_ (high) mode at about 437 cm^–1^ [22]. X-ray measurements were performed in a Bruker D8 Advance diffractometer (Billerica, MA, USA).

### 2.2. Nanofluid Synthesis

Oil-based nanofluids were prepared dispersing the nanoparticles in vegetable oil through an ultrasonic bath (Elmasonic P 300H, 80 kHz, Elma Schmidbauer GmbH) (Gottlieb-Daimler, Singen, Germany) for 7 h in intervals of 30 min of agitation and pause to refrigerate the nanofluid at room temperature avoiding overheating, to obtain stable colloids, which is critical for the preparation of dielectric nanofluids. Different concentrations of nanoparticles were prepared, however, only one of them (0.04 wt %) was used to carry out the aging study. The reason for that was that this concentration showed the highest increase of the breakdown voltage as it is detailed in Results section.

**Figure 5:**
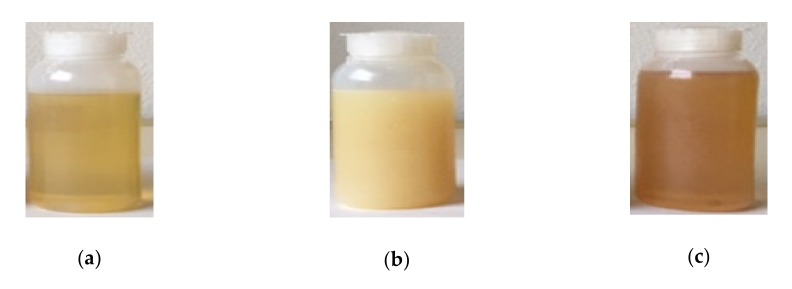
Oil samples: (**a**) natural ester; (**b**) natural ester + TiO_2_ nanoparticles (0.04 wt %) and (**c**) natural ester + ZnO nanoparticles (0.04 wt %).

A photography of nanofluids and base fluid analyzed during aging tests is shown in Figure 5.

Table 3 shows some of the initial properties of the vegetable oil (ester) and the nanofluids studied during aging in this work. These initial properties are the dissipation factor (tan δ) used to evaluate fluids’ performance as insulators; relative permittivity (ε) that is a diagnostic physical property, which characterizes the degree of electrical polarization a material experiences under the influence of an external electric field; specific resistivity (DC resistivity) applying a positive (+) and a negative (−) DC voltage utilized to indicate the existence of free ions, ion-forming particles and conductive contaminants and water concentration in the insulation liquid (moisture) and milligrams of KOH required to neutralize 1 g of the insulation liquid (acidity), this is a critical parameter for analyzing the oxidation of transformers’ oils.

Apart from the effect of the nanoparticles in the dielectric oil, an important aspect to consider is also the quality of their dispersion, since stable nanofluids would guarantee the correct performance of transformers. Most of the works in which the nanofluids stability was considered have applied visual inspection as a technique to evaluate the dispersion that has not aggregated [23]. This technique has been used by different authors to measure the stability period of nanofluids for different particle concentration and sonication time. The stability period is defined as the elapsed time after the preparation of nanofluids, at which aggregates are visible [24]. However, in this work the stability of the nanofluids over the time has been monitored through UV-Vis absorption spectroscopy, once the nanofluid had been produced, to carry out a more accurate study of stability. Absorption spectra as a function time were recorded using a double-beam spectrophotometer Cary 6000i, Agilent Technologies, Varian, Inc. (Santa Clara, CA, USA). The absorbance at two different wavelengths was evaluated as a function of time. The measurements were performed at room temperature without perturbing the liquid inside the 10 mm size cuvette during the whole experiment. In these experiments a cuvette with natural ester is used as reference in the double beam spectrometer. Additionally, the stability was also estimated through the average diameter of the nanoparticles suspended in the oil by means of the dynamic light scattering (DLS) technique. A Zeta sizer Nano S Malvern Panalytical, (Malvern, UK) was used to measure the nanoparticles size. This equipment can display the size from 0.3 to 10^4^ nm. The size measurement is based on a photon correlation, which is able to separate several different nanoparticle size populations in the test sample providing one peak for each of them. Only one peak means that almost all the nanoparticles have a diameter around the common mean value.

### 2.3. Methodology of Thermal Aging

An accelerated aging test was carried out in an oven at 150 °C for 312 h. This allowed the assessment of ageing effects on the nanofluids and on the Kraft paper. The limit of the temperature rise at the top of the oil in power transformers was set at 60 °C [25]. Therefore, an increase above this rise would elevate the aging rate as it happens with pure dielectric oils [14,15].

**Figure 6:**
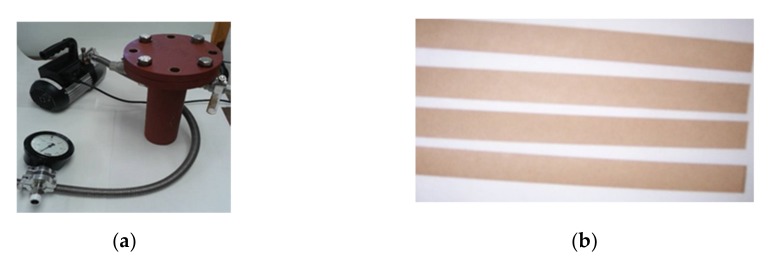
(**a**) Steel container and vacuum pump and (**b**) strips of Kraft paper.

In this sense the procedure followed in this work obeyed these steps:

- The dielectric fluids were introduced into steel containers where there were strips of Kraft paper (0.2 mm thickness; Figure 6).
- The Kraft paper was dried at 100 °C for 48 h to reduce its moisture content and the oil was degassed at 60 °C during 6 h.
- By means of a vacuum pump the air was extracted from the containers. Then the containers were filled with nitrogen.
- Finally, the steel containers were introduced into the oven at 150 °C.

Five samples of the vegetable oil and the nanofluid were taken to evaluate the progress of their aging at 150 °C. The thermal aging test of the nanofluid was compared with the carrier, vegetable oil, subjected to the same conditions as the nanofluid.

## 3. Results

Firstly, the selection of the optimum concentration of nanoparticles (TiO_2_ and ZnO) was done through breakdown voltage measurement. Secondly, the results of the measurements performed on the samples of dielectric nanofluids and base fluid (natural ester) during aging are summarized. These measurements include: stability, breakdown voltage, dissipation factor, resistivity, moisture and acidity. As for the paper condition, the analysis of its degradation was carried out through the measurement of the DP.

### 3.1. Optimum Concentration

**Figure 7:**
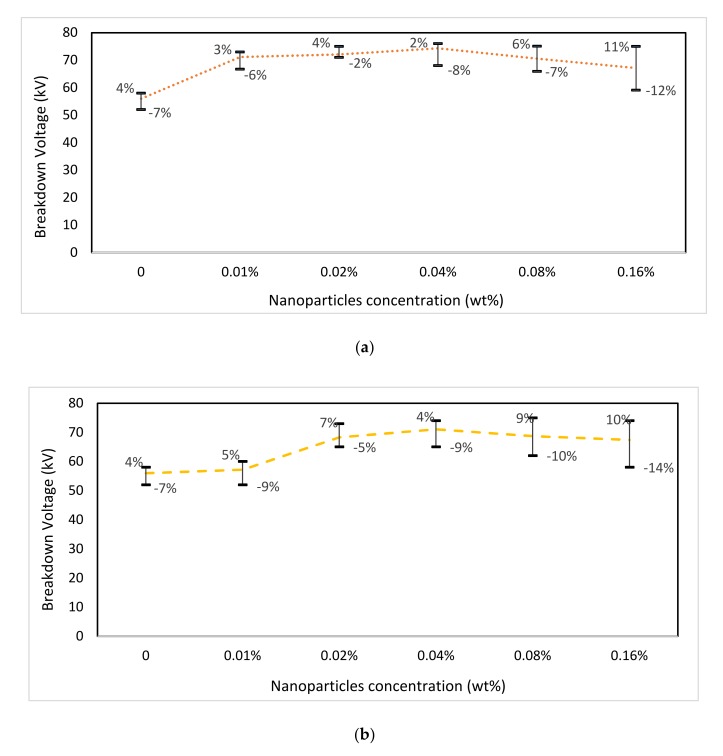
Breakdown voltage as function of particle concentration for (**a**) TiO_2_ nanoparticles and (**b**) ZnO nanoparticles.

The variation in the breakdown voltage of nanofluids with different weights of nanoparticles is gathered in Figure 7. This property was determined according to IEC 60156 [26] at 50 Hz.

It is observed that in both nanofluids the breakdown voltage increases with the addition of nanoparticles in natural ester as it has been shown by many other authors [27,28,29,30]. It was found that 0.04 wt % of TiO_2_ nanoparticles in the ester provides the highest breakdown voltage. In the case of ZnO nanoparticles, the results showed that there were two optimal concentrations, the first one was between 0.02 and 0.04 wt % and the second one between 0.04 and 0.08 wt %. These results revealed that before and beyond the two possible optimal concentrations, the dielectric performance of nanofluids falls, similar results have been obtained by several authors who have studied different nanoparticles [27,30]. In order to compare the behavior of both nanofluids during thermal aging under similar conditions, the nanofluids were aged with 0.04 wt % of both nanoparticles. In the case of ZnO nanoparticles, the lowest optimal concentration was selected because although it achieved a slightly lower breakdown voltage (−0.60%) than the maximum, it required a lower concentration of nanoparticles, reducing considerably the cost of obtaining the nanofluid.

It was also observed that the presence of TiO_2_ nanoparticles demonstrated higher dielectric performance with maximum breakdown voltage of 74.3 kV against a value of 71.1 kV obtained when ZnO nanoparticles were added to the ester.

Assuming that there is no chemical composition effect and the different behavior is only due to size differences, it can be concluded that the dielectric behavior is better for smaller nanoparticles with a larger surface area. This effect of the nanoparticles size on breakdown voltage has been explained by the equation of Brownian velocity [30,31], according to which smaller particles experience higher Brownian motion that is directly associated with the particles size and temperature. The Brownian velocity [31]:

(1) $$U_{B}=\left(\frac{2\cdot k_{B}\cdot T}{\pi \cdot \mu \cdot d_{np}^{2}}\right)$$

(where: *U_B_*, *k_B_*, *T* and *d_np_* are Brownian velocity, Boltzmann constant, absolute temperature, viscosity of pure oil and nanoparticles diameter respectively) is inversely proportional to the square of the particles size. Consequently, smaller nanoparticles will result in higher Brownian velocity, which ends up in higher disruption of the growth of the propagation of the breakdown streamer, thus higher breakdown voltage occurs at the end [32,33]. This is a simplified assumption and the size effect should be tested using the same material.

### 3.2. Nanofluid Stability

**Figure 8:**
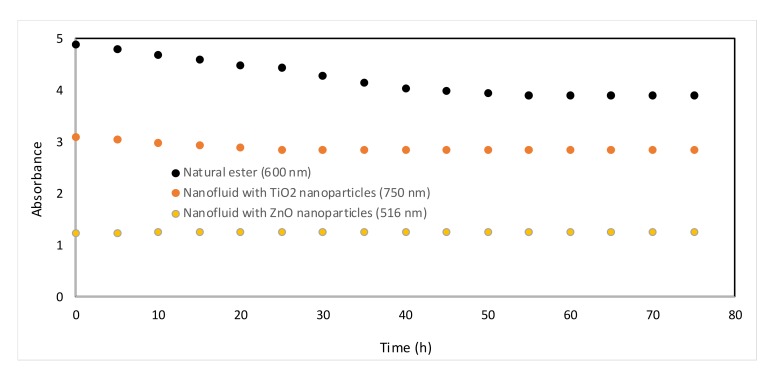
Stability analysis through absorbance measurement of natural ester and nanofluids.

Figure 8 shows the temporal stability of nanofluids produced by the sonication of ZnO and TiO_2_ nanoparticles in vegetable oil with 0.04 wt %, which was the nanofluid selected to carry out the aging study. The measurements were performed during at least 74 h after sonication. The longer wavelength was representative of the turbidity of the nanofluids, whereas the shorter one was related to the tail of the energy gap of the fundamental absorption of the wide bandgap semiconductor. Although the ZnO nanofluid was more stable than that produce with TiO_2_ both nanofluids were stable in time. In both cases the nanofluids reach a steady state much higher than the reference value (vegetable oil).

**Figure 9:**
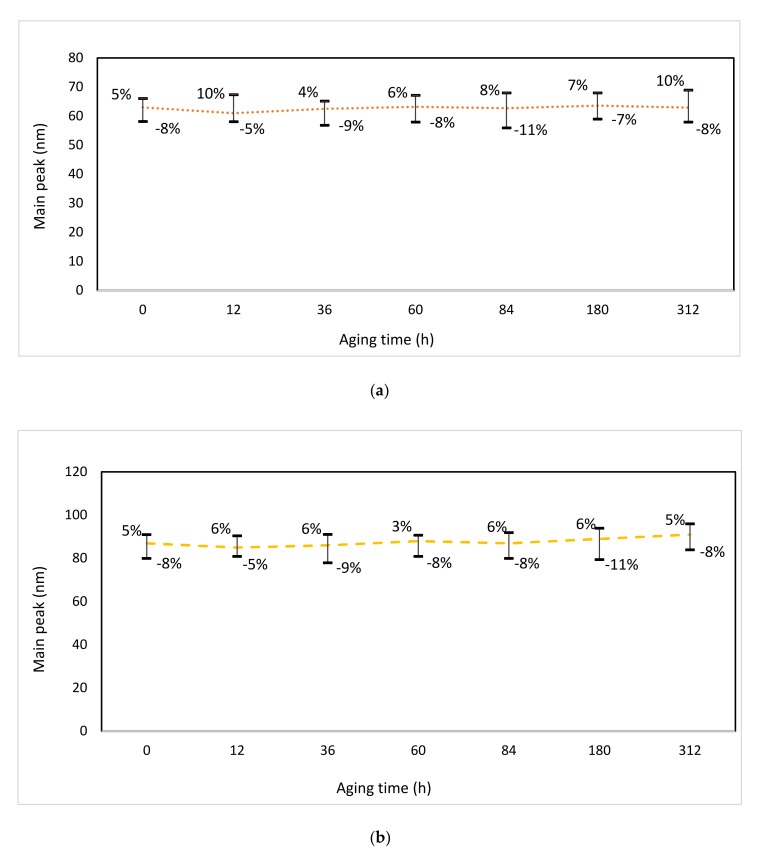
Average nanoparticle size during thermal aging: (**a**) TiO_2_ and (**b**) ZnO.

As it was detailed previously, the stability was also estimated through the average diameter of the nanoparticles suspended in the insulation oil by means of the DLS technique (Figure 9).

As can be observed the particle-size is higher than that obtained through TEM images at the beginning of the aging. That is because DLS transforms a diffusion coefficient to an equivalent hydrodynamic diameter, which is the size of the nanoparticle plus the liquid layer around the particle, whereas TEM measures the actual size of the nanoparticle.

Although the visual inspection of both nanofluids seemed to indicate that they remained stable for the aging period, in the case of the nanofluid with ZnO nanoparticles there is a slight increase on the particle size as the testing time rises. Therefore, it might be deduced that the formation of aggregates increases with time at high temperatures, being observed that this effect starts before when particle size is upper. Subsequently, it could result in a critical problem if nanofluids are implemented in power transformers operating with high hot-spot temperatures.

### 3.3. Density and Viscosity

The addition of nanoparticles in the oil modifies its viscosity [34,35], which is a measurement of the resistance of a fluid to flow. This physical property has high influence in thermal performance of transformer oils [36] because the lower is the viscosity, the better the power transformer cooling. Another property that has effect on heat dissipation is the density. For these reasons, it was studied the evolution of oil density and viscosity with and without nanoparticles at different temperatures.

**Figure 10:**
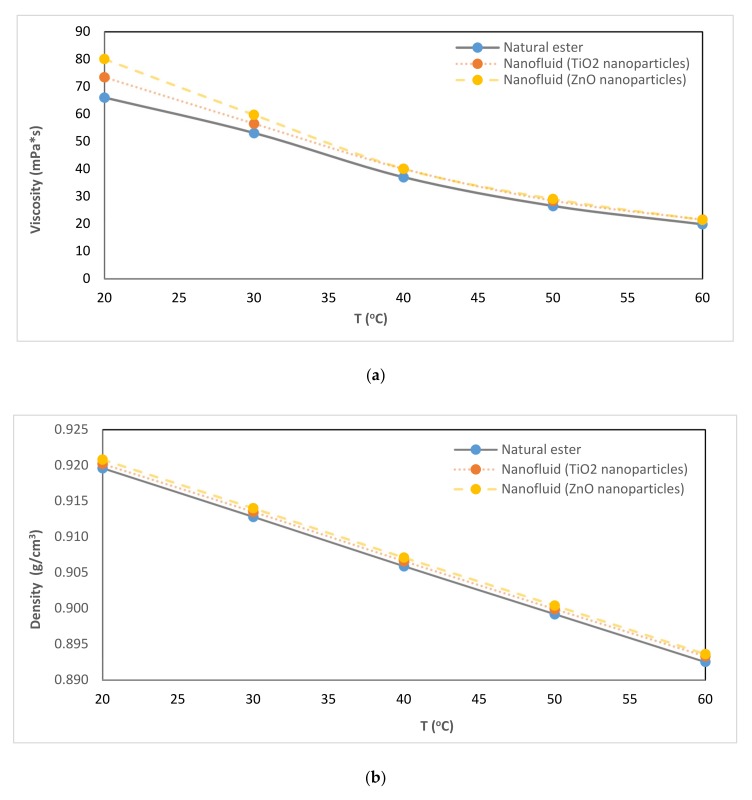
Evolution with temperature of (**a**) viscosity and (**b**) density.

It was observed that viscosity decreases with an increase in temperature in both nanofluids and in the natural ester. The results have shown that the viscosity behavior of pure oil with temperature is very similar to that shown by nanofluids in the temperature range of 40–60 °C, as can be seen in Figure 10. However, under a lower temperature range (20–30 °C) it was clearer that an increase in viscosity of the nanofluids with nanoparticles dispersed in the transformer oil. The presence of TiO_2_ nanoparticles increased the viscosity of the fluid in 11.2% at 20 °C. This increase was 21.3% when ZnO nanoparticles were used to obtain the nanofluid. These results were similar to those obtained by other authors who have found that not much variation in this property is obtained when low concentrations of nanoparticles are dispersed in transformer oils [23,27].

Regarding the density, its behavior with temperature was very similar to that shown by nanofluids in the temperature range of 20–60 °C.

### 3.4. Breakdown Voltage

The breakdown voltage test is generally used as an acceptance test before filling the oil inside the transformers. This test serves to decide on the possibility of carrying out a drying and filtration treatment because its value depends on the moisture content and other impurities present in the sample. In this work the breakdown voltage was performed using stainless steel electrodes, which were set 2.5 mm apart and according to IEC 60156 [26].

**Figure 11:**
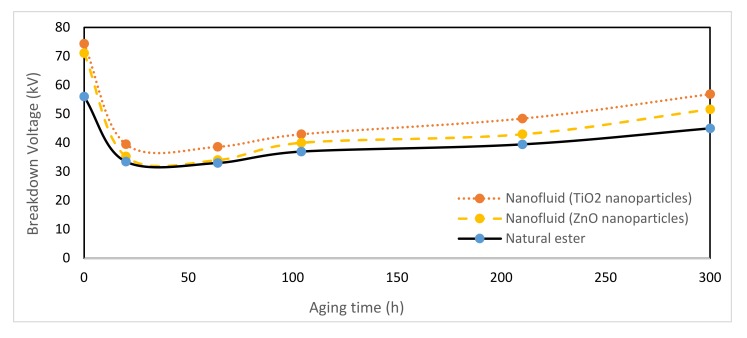
Evolution of breakdown voltage over time at 150 °C.

In the laboratory experiments, it was observed, in Figure 11, that the addition of nanoparticles in dielectric vegetable oil generally improved the dielectric strength, especially from 100 h of aging. Moreover, the TiO_2_ modified oil reached even higher value than ZnO modified oil. At the end of the aging period, it has been obtained that both nanofluids based on ZnO and TiO_2_ nanoparticles possesses better dielectric strength than vegetable oil. The values of nanofluids’ breakdown voltage were 28% (TiO_2_) and 15% (ZnO) higher than vegetable oil.

Additionally, it is needed to highlight the fact that initially this dielectric property suffers a considerable decrease in the three dielectric liquids. Nevertheless, once a period of 20 h has passed this trend is reversed. This behavior can be explained through the evolution of moisture in the dielectric fluids. As it will be seen below, at the beginning of the accelerated thermal aging there was a significant increase in the oil’s moisture, which adversely affected the dielectric strength. Therefore, whenever the moisture of the oil decreased, dielectric strength underwent a rise despite of increasing aging time. Although it may appear that these results were not in harmony with the behavior of dielectric strength during aging, they were, because when the values of dielectric strength were compared in samples with similar moisture, which was decreasing considerably during the aging period, it was found that the breakdown voltage decrease due to thermal aging. For example, if the breakdown voltage of the new TiO_2_ nanofluid (t = 0 h) was compared with the one obtained when this liquid has been aged for 75 h (at this time the moisture of the nanofluid was similar to the one at the beginning), the nanofluid had reduced its breakdown voltage by 50% approximately of its initial value, that is, the aging causes a reduction in dielectric strength.

### 3.5. Moisture

**Figure 12:**
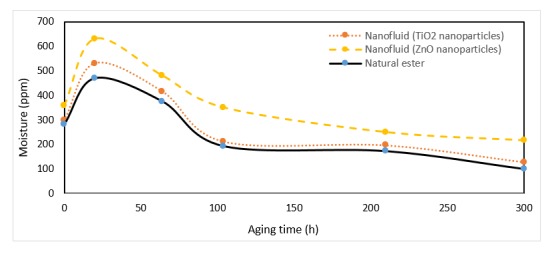
Evolution of moisture in dielectric fluids over time at 150 °C.

**Figure 13:**
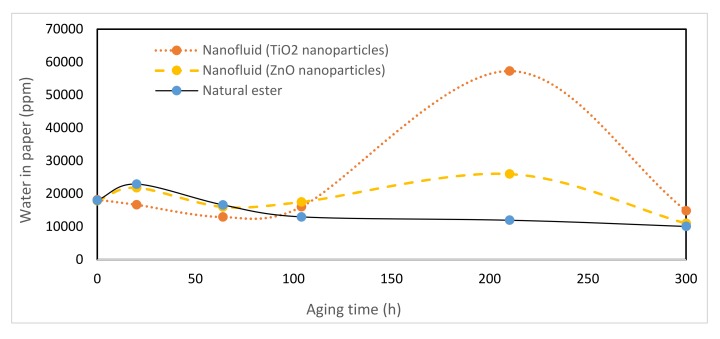
Evolution of moisture paper over time at 150 °C.

Water moved between the paper insulation and the oil trying to achieve equilibrium terms of relative saturation, it is also known that natural esters can accommodate more water than conventional transformer oil [37,38]. This difference causes equilibrium transference toward the vegetable oil [39], thus more water is moved from the paper to the oil [40]. Analyzing the results of moisture measurement [41], it can be observed that the moisture content in the nanofluid is slightly higher than that of the vegetable oil for most of the aging period (Figure 12). While the moisture in the paper was lower with nanofluids, with the same initial moisture, Figure 13.

It was observed that the moisture of nanofluids and the ester during the first 50 h of aging was increasing. From this time on moisture concentration diminished in all the liquids, showing the nanofluids higher water content during the whole aging period.

Analyzing the behavior of these samples, an important increase of the moisture in the early stages of the process was verified. This is caused because water is a byproduct of oil and paper degradation, which seems to be enhanced by the presence of particles. Subsequently, this content decreased gradually, reaching similar values in both fluids, less than 220 ppm at the end of aging.

On the other hand, the moisture in the paper immersed in the nanofluids decreased during the first 100 h, Figure 12. Since that moment the moisture in the paper increased up to 200 h in nanofluids while in the paper impregnated by vegetable oil the moisture was kept constant its value being around 10%.

The significant reduction of moisture in the dielectric fluids from 64 to 100 h of aging helped to explain the increase in dielectric strength despite having more degraded liquids.

### 3.6. Dissipation Factor

**Figure 14:**
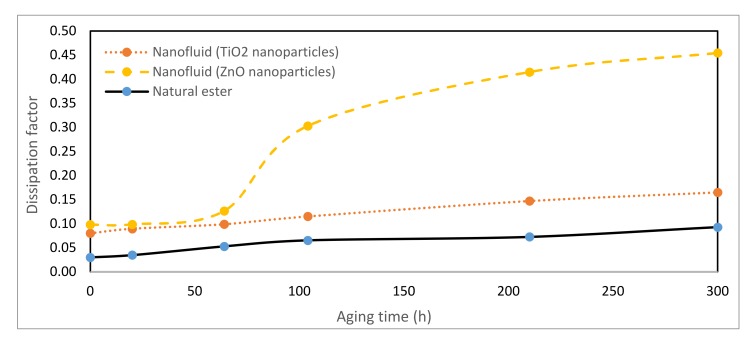
Evolution of dissipation factor over time at 150 °C.

The dielectric dissipation factor is a suitable tool to indicate the quality of insulation. A high value of this parameter is an indication of the presence of contaminations or deterioration products such as water or oxidation products. Figure 14 shows the evolution of the dissipation factor, determined according to IEC standard [42], during the thermal aging of the vegetable oil and the nanofluids. It can be observed that the nanofluids presented a dielectric dissipation factor higher than that of the vegetable oil.

The dissipation factor of the nanofluids is worse than that of the pure ester during the whole aging period. However, the nanofluid based on TiO_2_ nanoparticles had a behavior more similar to that of the natural ester, whereas the results show that the second nanofluid suffered a significant increase of its dissipation factor, obtaining a huge difference with respect to the vegetable fluid at the end of the thermal aging. These results verified the adverse effect of nanoparticles on the dissipation factor of the studied vegetable oil.

### 3.7. Resistivity

The resistivity of an insulation material is the ratio between the intensity of a continuous electric field and the steady state value of the current density in the material and it is the most sensitive property of oil requiring the utmost care for its proper determination. A low value indicates the presence of moisture and conductive contaminants.

**Figure 15:**
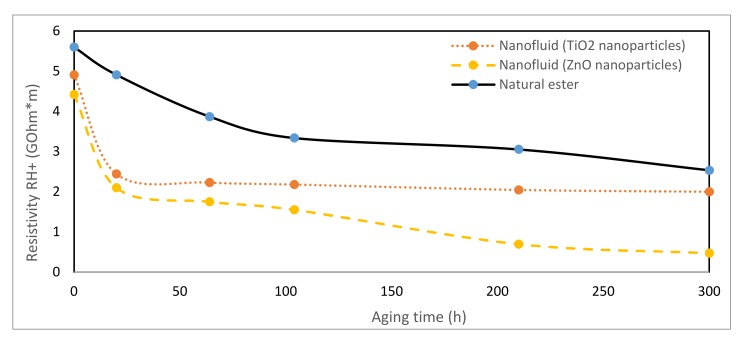
Evolution of resistivity (+) over time at 150 °C.

**Figure 16:**
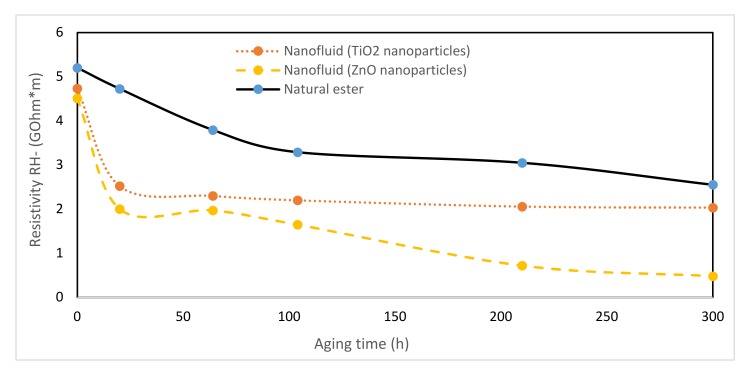
Evolution of resistivity (−) over time at 150 °C.

This parameter was measured [42] during thermal aging for both dielectric nanofluids and the vegetable ester. Positive and negative polarities were analyzed, obtaining quite similar results (Figure 15 and Figure 16). The nanofluids always presented lower resistivity than that of vegetable oil during aging. Analyzing the trends, it was observed that as the TiO_2_ nanofluid degraded, its resistivity was closer to the vegetable oil one.

### 3.8. Acidity

Acidity is a property whose value is a consequence of the byproducts of dielectrics degradation. In turn, this property is also one of the causes that favor the degradation of insulation systems and this is the reason why its value is limited in those fluids of dielectric application. It was determined according to IEC Standard [43].

**Figure 17:**
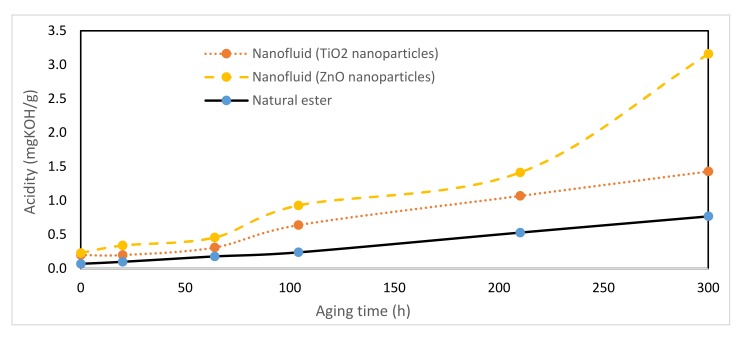
Evolution of acidity over time at 150 °C.

As with the dissipation factor, the nanofluids had a higher acidity than vegetable oil, Figure 17, which produced a greater degradation of the oil. Therefore, the presence of nanoparticles catalyzed the degradation of dielectrics, which was harmful for the insulation paper reducing the lifespan of the insulation system of power transformers.

### 3.9. Degree of Polymerization

The degree of polymerization (DP) can be defined as the average number of monomers present in a cellulose molecule. This property is an indirect measurement of the mechanical strength of the paper and therefore of its state of degradation. This property was measured according to IEC standard [44].

**Figure 18:**
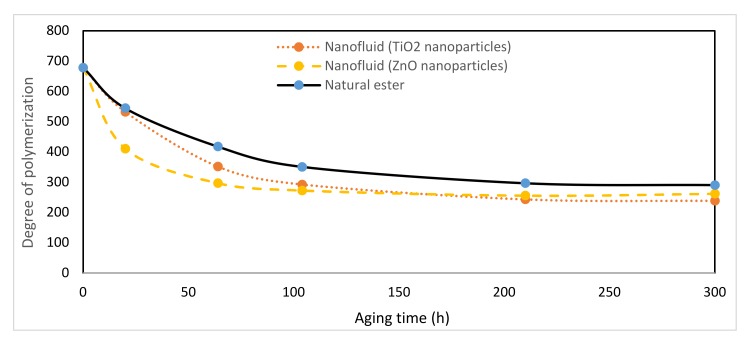
Evolution of degree of polymerization over time at 150 °C.

Figure 18 shows the evolution of the DP throughout the aging period, there was general agreement that temperature was the main factor influencing the aging rate of the solid insulation within transformers. At least two determinations of the DP were made on each paper sample extracted. These results corroborate that the values of DP corresponding to the Kraft paper aged in the ZnO nanofluid could be reduced up to 30% with respect to the Kraft paper aged with the pure vegetable oil, at the beginning of aging period. Later the DP values of both fluids tended to be very similar. Therefore, the nanoparticles raised the degradation rate of Kraft paper especially at the first stages of the degradation process.

## 4. Conclusions

This paper evaluated the effect of TiO_2_ and ZnO nanoparticles on the aging of an insulation system based on a natural ester and Kraft paper.

It could be concluded that nanoparticles increased the values of ageing indicators such as dissipation factor and acidity, as well as reduced the degree of polymerization of dielectric paper, which indicates higher degradation of paper.

The dissipation factor measurement is normally used to observe the effect of the products of oil degradation on its dielectric capacity. In this work the influence of nanoparticles on the degradation of the dielectric capacity of oils was also studied. It was observed that this degradation fulfilled the usual limits, regulated by the IEC 60422 for mineral oil. There is currently no regulation on the limits that vegetable oils that are in a transformer in operation should meet. For this reason, the obtained results were compared with the dissipation factor standard for mineral oils.

A possible explanation for this higher degradation, which causes more acidity, a higher dissipation factor and lower resistivity, may be that the nanoparticles act as hot spots, promoting the thermal degradation of the dielectrics. However, the use of nanoparticles increased the value of breakdown strength, leading to the existence of an optimum concentration of particles. This is essential for a suitable performance of power transformers.

A critical issue observed at the end of the aging period, in the nanofluid obtained with ZnO nanoparticles, was a slight increase of the average size of particles. This behavior was determined using DLS. This slight increase might represent the beginning of nanoparticles agglomeration ending up in the loss of nanofluids stability. Consequently, more research is still required to guarantee long-term stability of these liquids during transformers operation.

## Figures and Tables

**Table 1:** Specifications of vegetable oil used for the nanofluids.

Property	Specification
Breakdown Voltage kV	>75
Kinematic Viscosity at 40 °C	37
Density at 20 °C	0.92
Pour point °C	−31

**Table 2:** Specifications of nanoparticles.

Property	Specification ZnO	Specification TiO_2_
Density (g/cm^3^)	5.61	3.89
Specific surface area (m^2^/g)	>50	>120
Purity (%)	99.5	99.5
Average diameter (nm)	60	45

**Table 3:** Characteristics of fluids without aging.

Fluid	Characteristics					
	tan δ-	ε-	DC Resistivity		Moisture	Acidity
			GΩ-m (+)	GΩ-m (−)	ppm	mg KOH/g
Ester	0.030	2.80	5.60	5.20	281	<0.07
Ester + ZnO	0.098	2.80	4.42	4.51	358	0.23
Ester + TiO_2_	0.080	2.80	4.91	4.73	297	0.2
